# Not "just" an intravenous line: Consumer perspectives on peripheral intravenous cannulation (PIVC). An international cross-sectional survey of 25 countries

**DOI:** 10.1371/journal.pone.0193436

**Published:** 2018-02-28

**Authors:** Marie Cooke, Amanda J. Ullman, Gillian Ray-Barruel, Marianne Wallis, Amanda Corley, Claire M. Rickard

**Affiliations:** 1 Alliance for Vascular Access Teaching and Research (AVATAR), Menzies Health Institute Queensland, Griffith University, Brisbane, Australia; 2 School of Nursing and Midwifery, Griffith University, Brisbane, Australia; 3 School of Nursing, Midwifery and Paramedicine, University of the Sunshine Coast, Sippy Downs, Australia; University of Liverpool, UNITED KINGDOM

## Abstract

Peripheral intravascular cannula/catheter (PIVC) insertion is a common invasive procedure, but PIVC failure before the end of therapy is unacceptably high. As PIVC failure disrupts treatment and reinsertion can be distressing for the patient, prevention of PIVC failure is an important patient outcome. Consumer participation in PIVC care to prevent failure is an untapped resource. This study aimed to understand consumers’ PIVC experience; establish aspects of PIVC insertion and care relevant to them; and to compare experiences of adult consumers to adult carers of a child. An international, web-based, cross-sectional survey was distributed via social media inviting adult consumers and adult carers of a child under 18 years who had experienced having a PIVC in the last five years (one survey each for adults and adult carers) to complete a 10-item survey. As such, sampling bias is a limitation and results should be carefully considered in light of this. There were 712 respondents from 25 countries, mainly female (87.1%) and adults (80%). A little over 50% of adults described insertion as moderately painful or worse, with level of insertion difficulty (0–10 scale) identified as moderate (median 4, IQR 1, 7). Adult carers reported significantly more pain during insertion and insertion difficulty (both p < 0.001). Rates of first insertion attempt failure were higher in children compared with adults (89/139 [64%] vs 221/554 [40%]; p < 0.001), and 23% of children required ≥ 4 attempts, compared with 9% of adults (p < 0.0001). Three themes from open-ended question emerged: Significance of safe and consistent PIVC care; Importance of staff training and competence; and Value of communication. The PIVC experience can be painful, stressful and frustrating for consumers. Priorities for clinicians and policy makers should include use of pain relief as standard practice to reduce the pain associated with PIVC insertion and developing strategies to increase first PIVC insertion attempt success particularly for children and older consumers.

## Introduction

Peripheral intravascular cannula/catheter (PIVC) insertion is the most widely performed invasive procedure in hospitals with up to 70% of inpatients requiring a PIVC during their stay [1]. However, rates of PIVC failure and unscheduled restarts are unacceptably high, with rates ranging from 33% to 69% [2–6]. Reasons for PIVC failure include accidental removal or dislodgement, pain, phlebitis, occlusion, infiltration and infection [3, 6, 7]. PIVC failure and related complications are costly to both the health care system and the consumer. With each failure, human and material resources are required to re-site the PIVC so treatment can continue. The cost of treating complications associated with PIVC failure also must be considered. The burden of PIVC failure on consumers is overlooked or underemphasized in the literature and by clinicians [8]. PIVC re-sites can be painful and distressing, with frequent cannulation attempts adversely affecting the person’s overall hospital experience [8]. In the paediatric population, PIVC placement in hospital is self-reported as the leading source of procedure-related pain [9–11].

Few studies report consumers’ experiences of PIVC insertion and care [12]. Some research on the experiences in relation to other vascular access devices (for example, peripherally inserted central catheters [13–15], totally implanted central venous devices [16, 17]) or in relation to certain patient populations (for example, cancer [18, 19]; or renal [20] patients) can be found but these are limited. Such previous research primarily focused on the psychosocial issues surrounding having a vascular access device, about catheter-related infections and the pain associated with intravascular devices, but few examined consumer perspectives about the aspects of PIVC insertion and care relevant and important to them.

In vulnerable populations, such as older people with multiple comorbidities, or neonates and young people, an emphasis must be placed on first attempt PIVC insertion success as the PIVC placed on first attempt is the least likely to subsequently fail from complications [21]. Strategies to improve PIVC care and functional dwell time, such as new PIVC designs, advanced dressings and securement devices [22], flushing techniques [23], and PIVC care bundles [24], have been tested as a means of reducing PIVC failure rates with some promising outcomes; however, the role of the consumer in mitigating PIVC failure has not been explored to date. Consumers could be a powerful and untapped tool in the decision-making process related to insertion and care of vascular access devices (VAD), such as peripheral intravascular cannulas, to minimise the risk of complications and failure. One US study identified that patients who had high levels of participation in care were half as likely to experience an adverse event, compared to those with low participation [25]. About 40% of patients are unaware why they have a VAD and a similar number have an unnecessary VAD [26]. Patients’ lack of awareness of the reason for their PIVC has been associated with a 7-fold increase in unnecessary PIVCs [26].

Consumer participation and engagement is a core aspect referred to in safety and quality health service standards around the world [27–29]. However, rigorous studies examining the impact of patient-clinician partnerships on patient outcomes in the acute care setting are lacking. VAD management is increasingly focused on bundled interventions for insertion and management [30], and consumer participation in such bundles is a potentially cost-effective strategy to reduce VAD complications and improve outcomes.

Understanding consumers’ perspectives of the PIVC insertion and care experience is key to establishing strategies to engage them in the care of their PIVC. Therefore, the aim of this study was to understand consumers’ experience of a PIVC for therapy, and to establish what aspects of PIVC insertion and care are relevant and important to them. Additionally, the study aimed to highlight differences in the experiences of the two targeted consumer groups: adults with a PIVC and adult carers of a child (ACC) with a PIVC. As such, findings from the study will inform the inclusion of patient reported outcomes and strategies for consumer participation and engagement in care in future research and translation of research.

## Material and methods

Ethical approval was gained from Griffith University’s Human Research Ethics Committee (GU Ref No: 2016/001) before the study commenced. An international, web-based, cross-sectional survey was undertaken to establish consumers’ PIVC experiences. An invitation to complete an on-line, anonymous and voluntary survey was distributed via the research group’s (AVATAR [Alliance for Vascular Access Teaching and Research]) social media (Twitter and Facebook) accounts. The survey was open from March to November 2016. Adults over the age of 18 who had experienced having a PIVC in the last 5 years were invited to complete the survey. To capture paediatric experiences, ACC under 18 years of age who had experienced having a PIVC in the last 5 years were also invited to complete a survey. The invitations included a link to both on-line surveys. We have used the Reporting of Observational studies in Epidemiology (STROBE) guidelines to report this study.

Data were collected via the Griffith University’s Survey Centre (LimeSurvey™ LimeSurvey GmbH, Hamburg, Germany. URL http://www.limesurvey.org), which provides a secure environment for data storage. An information sheet containing an invitation to participate and details of the study was provided on the web-page and a completed survey was taken as a sign of consent. Both surveys consisted of 10 questions. The survey questions were developed from topic areas identified from a review of the literature and then distributed to five senior members of the AVATAR group with research and clinical expertise in vascular access. The questions then went through three rounds of discussion until agreement was reached. Revisions included changes to promote greater clarity of items and to ensure choices for responses to some questions were appropriate.

### Statistical analysis

Descriptive analyses were used for Questions 1–9 to provide percentages and medians (IQR). To compare the PIVC experiences of adult consumers and ACCs, the Mann-Whitney U test, Chi-square or Fisher’s exact test were used for categorical data. P-values of < 0.05 were considered statistically significant. A thematic analysis of responses from the last open-ended question was completed, based on Norwood’s approach [31]. The pattern of categories and the relationships between categories were identified from a manual coding of the responses and systematically considered using an inductive analytic process to allow themes to emerge from the data. Thematic names were chosen based on their clarity to represent the overall sense of the respondents’ comments.

## Results

**Fig 1:**
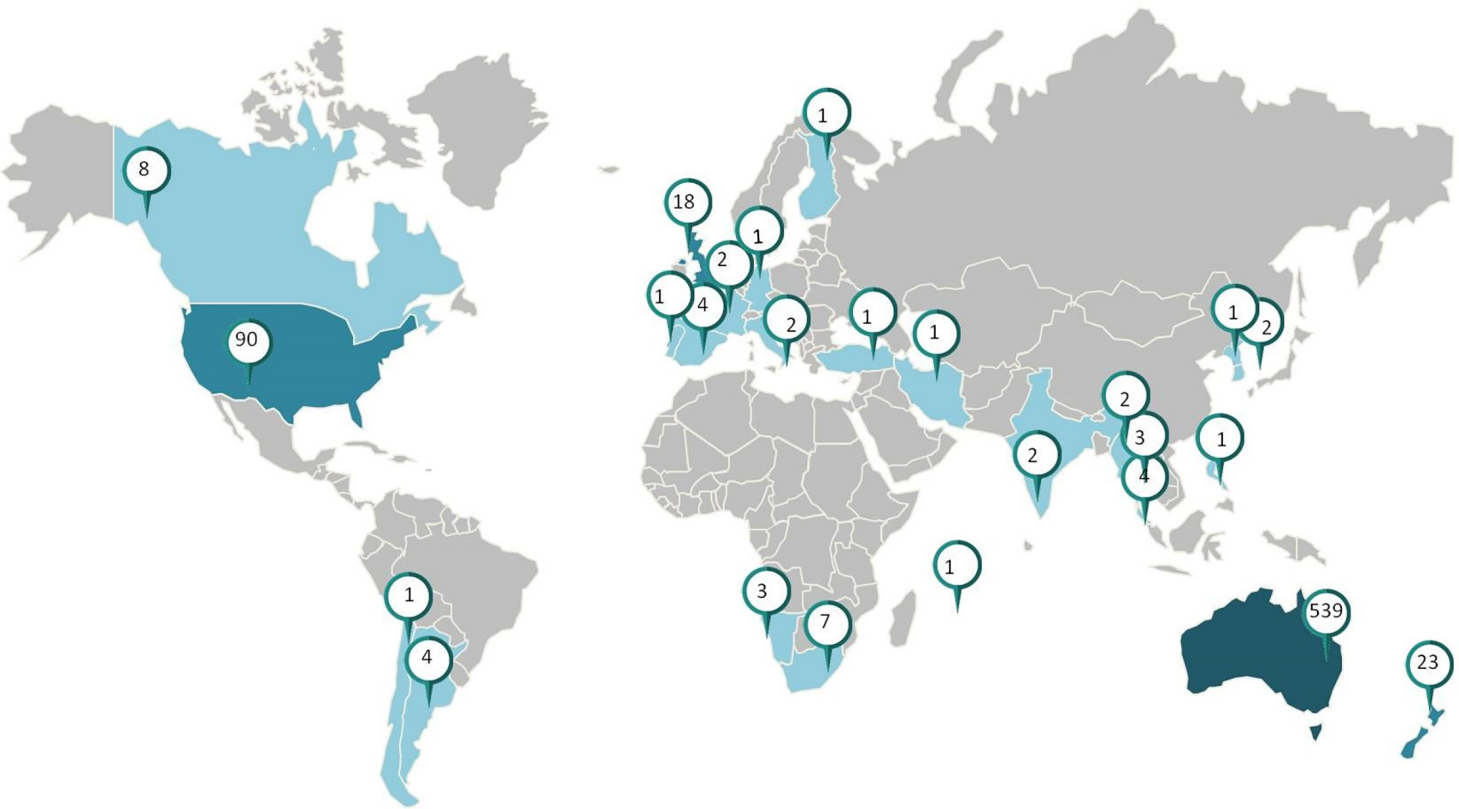
Geographical distribution of respondents.

There were 712 respondents to the online survey, mainly female (87%) and from Australia (74%). Adult consumers comprised 80% of the cohort and ACC made up the remainder (20%) of respondents. Table 1 outlines the demographics of respondents and Fig 1 represents the geographical distribution.

**Table 1:** Demographics of survey respondents and subjects (n = 712).

		Adult survey	Paediatric survey
		(n = 570)	(n = 142)
**Female sex**	n (%)	492 (66.7)^a^	128 (90.1)^c^
**Female sex of child**	n (%)	-	68 (47.9)^d^
**Age of respondent (years)**	Mean (sd)	44 (12.9)^b^	38.5 (8.6)
**Age of child (years)**	Mean (sd)	-	6.3 (5.0)
**Country of residence**	Australia	420 (73.7)^b^	109 (76.8)
n (%)	United States	74 (12.9)	16 (11.3)
	New Zealand	22 (3.8)	1 (0.7)
	United Kingdom	12 (2.1)	6 (4.2)
	South Africa	6 (1.0)	1 (0.7)
	Canada	5 (0.9)	3 (2.1)
	Spain	3 (0.6)	1 (0.7)
	Argentina	3 (0.5)	1 (0.7)
	Namibia	3 (0.5)	0
	Singapore	3 (0.5)	1 (0.7)
	Malaysia	2 (0.3)	1 (0.7)
	France	2 (0.3)	0
	India	2 (0.3)	0
	South Korea	2 (0.3)	0
	Thailand	2 (0.3)	0
	Italy	0	2 (1.4)
	Chile	1 (0.2)	0
	Finland	1 (0.2)	0
	Germany	1 (0.2)	0
	Iran	1 (0.2)	0
	North Korea	1 (0.2)	0
	Philippines	1 (0.2)	0
	Portugal	1 (0.2)	0
	Reunion	1 (0.2)	0
	Turkey	1 (0.2)	0

Missing data

^a^2

^b^1

^c^1

^d^2

Table 2 contains the responses from adult consumers and ACCs to the survey questions regarding PIVC insertion. From the cohort of adults describing their experience, over 50% of respondents had six or more PIVCs in the previous five years and described the insertion experience as moderately painful or greater (on a scale of 0–10, median 4, interquartile range (IQR) 2, 7) and the mean level of difficulty in inserting the PIVC (on a scale of 0–10) as moderate (median 4, IQR 1, 7). Difficult PIVC insertion was reported by nearly 51% of respondents and 28% of these respondents believed that the difficulty could be attributed to insufficient skill of the inserter. However, nearly 60% of respondents believed that the inserter of their PIVC was well trained. The determinants of a successful PIVC insertion were reported to be well-trained staff (89%), good hydration (63%), good arm position (43%), heat compress (32%) and ultrasound guidance (29%). The responses from adult consumers showed that age was a significant factor in both difficulty of insertion and pain or stress associated with insertion, with older respondents (i.e. > 65yrs) experiencing more difficult (p = 0.001; one way ANOVA) and painful insertions (p = 0.049; one way ANOVA) than middle age or younger respondents.

**Table 2:** Adult and paediatric survey: PIVC insertion.

Questions	Adult Survey	n (%)	Paediatric Survey	n (%)	p value
	Responses		Responses		
	n = 570		n = 142		
**Number of previous IVs**^a^	1	57 (10)		30 (21.1)	
	2–5	221 (38.8)		51 (35.9)	
	6–10	123 (21.6)		14 (9.8)	
	> 10	167 (29.3)		47 (33.1)	
**How much difficulty did health staff have when trying to insert an IV cannula into your/your child’s veins?** ^b^	**Median (IQR)**	4 (1, 7)		7 (3, 8)	*<0*.*001^*
*1 = not difficult;*	No–minimal difficulty (≤ 3)	278 (48.8)		45 (31.7)	
*10 = very difficult*	Moderately difficult (4–7)	168 (29.5)		37 (26.0)	
	Difficult (≥ 8)	122 (21.4)		60 (42.2)	
**(If 4 or more): why do you think the insertion was difficult?**	Difficult veins	170 (43.9)	Child’s difficult veins	53 (54.6)	
	Personnel skill	109 (28.2)	Child’s young age	39 (40.2)	
*Multiple responses per*	Staff difficulty inserting cannula	114 (29.5)	Staff difficulty inserting cannula	42 (43.3)	
*participant*	Difficult to feel veins	107 (27.6)	Difficult to see veins	40 (41.2)	
	Difficult to see veins	96 (24.8)	Difficult to feel veins	37 (38.1)	
	Underlying health	66 (17.1)	Personnel skill	28 (28.9)	
	Needle phobia	10 (2.6)	Underlying health	28 (28.9)	
	Dehydration	4 (1.3)	Child was moving	19 (19.6)	
	Other	17 (5.9)	Needle phobia	12 (12.4)	
			Dehydration	1 (1.0)	
			Other	6 (6.0)	
**What do you think helps in inserting the IV successfully?**	Well trained staff	507 (88.9)	Well trained staff	120 (84.5)	
	Hydration	361 (63.3)	Hydration	68 (47.9)	
*Multiple responses per*	Arm position	247 (43.3)	Equipment to see vein	58 (40.8)	
*participant*	Heat compress	185 (32.4)	Topical anaesthetic	51 (35.9)	
	Equipment to see vein	167 (29.3)	Distraction	48 (33.8)	
	Smaller cannula	131 (22.0)	Arm position	46 (32.4)	
	Distraction	70 (7.0)	Heat compress	29 (20.4)	
	Topical anaesthetic	61 (10.7)	Smaller cannula	29 (20.4)	
	Other	32 (5.6)	Other	17 (11.0)	
**How well trained do you think the person who inserted your/ your child’s most recent IV was?**^c^	**Median (IQR)**	8 (5, 10)		8 (6, 9)	0.44*^*
*1 = not well trained*	Not well trained (≤ 3)	72 (12.8)		12 (8.9)	
*10 = well trained*	Moderate (4–7)	156 (27.6)		43 (31.8)	
	Well trained (≥ 8)	336 (59.6)		80 (59.2)	
**Last time you/your child needed an IV, how many attempts were made to insert the IV?**^d^	1	310 (55.9)		37 (26.6)	
	2	95 (17.1)		23 (16.5)	
	3	79 (13.9)		33 (23.7)	
	4	20 (3.5)		11(7.9)	
	5	8 (1.4)		5 (3.6)	
	>5	23 (4.1)		17 (12.2)	
	I don’t remember	9 (1.6)		3 (2.1)	
**Last time you/your child needed an IV, how painful or stressful was the experience?** ^e^	**Median (IQR)**	4 (2, 7)		7 (5, 9)	*<0*.*001^*
*1 = no pain/distress;*	Minimal pain/distress (≤ 3)	268 (47.5)		25 (18)	
*10 = extreme pain/distress*	Moderate (4–7)	197 (34.9)		45 (32.4)	
	Severe pain/distress (≥ 8)	99 (17.5)		69 (49.6)	

IV = intravenous catheter. IQR = interquartile range.

Missing data

^a^ adult survey = 2, paediatric survey nil

^b^ adult survey = 2, paediatric survey nil

^c^ adult survey = 6, paediatric survey = 7

^d^ adult survey = 16, paediatric survey = 3

^e^ adult survey = 6, paediatric survey = 3.

Statistical tests

^Mann-Whitney U test

Children had slightly fewer previous PIVCs than adults, with 43% of children having six or more PIVCs in the last five years. Difficult PIVC insertion was reported by over two thirds (68%) of ACCs and, of these 68%, contributing factors to difficult PIVC insertion were identified as the child’s difficult veins (55%), the child’s young age (40%) and staff difficulty in inserting a cannula (43%). ACCs also rated well trained staff (84%) and hydration (48%) to be the most important factors in successful PIVC insertion, however, they rated ultrasound guidance (41%) and topical anaesthesia (40%) higher than adults did with a PIVC (Table 2).

ACCs reported significantly more difficulty with insertion and a more painful and stressful experience when compared with adults (both p < 0.001). Similarly, ACCs reported more complications with their child’s most recent PIVC when compared with adults (33.8% vs 25.7% respectively; *X*^2^ = 3.56; p = 0.059). The most common complications described by adults were bruising (12%), pain (10%) and swelling (9%) at the insertion site. In contrast, children experienced significantly more episodes of fluid leaking from the PIVC site; PIVC no longer working; PIVC no longer being in the vein; and PIVC dislodgement. The number of first insertion attempt failures was significantly higher in children when compared with adults (89/139 vs 221/554; p < 0.001). Consequently, more PIVC insertion attempts were made on children, with 23% of children requiring ≥ 4 attempts compared with 9% of adults (*X*^2^ = 22.3; p < 0.0001) (Table 3).

**Table 3:** Comparison of adult and paediatric PIVC experiences: PIVC insertion, post insertion issues, and complications (n = 712).

		Adult	Paediatric	P-value
		(n = 570)	(n = 142)	
		N (%)	N (%)	
**Last time you/your child needed an IV, did you have any cause for concern?**	No	363 (66.6)^a^	77 (56.2)^b^	**0.023**
	Pain at site	112 (19.6)	32 (21.0)	0.444
*Multiple responses per participant*	Difficulties washing/eating	54 (9.5)	18 (12.7)	0.257
	Catch things when mobilising	46 (8.1)	24 (16.9)	**0.002**
	IV pump alarm	40 (7.0)	20 (14.8)	**0.007**
	Child tried to move IV	-	14 (.2)	**-**
	Dressing loose	25 (4.4)	9 (6.3)	0.329
	Dressing wet or dirty	10 (1.7)	5 (3.5)	0.195^x^
	Other	60 (10.5)	18 (11.8)	0.879
**Did you/your child experience any complications with your most recent IV?**	No	403 (74.3)^c^	86 (66.2)^d^	0.059
	A lot of bruising around site	69 (12.1)	14 (9.8)	0.456
*Multiple responses per participant*	IV was too painful	58 (10.2)	17 (12.0)	0.533
	Swollen	51 (8.9)	16 (11.3)	0.397
	IV stopped working	42 (7.4)	25 (17.6)	**<0.0001**
	IV no longer in vein	26 (4.6)	16 (11.3)	**0.002**
	Very red around IV site	27 (4.7)	11 (7.7)	0.153
	Fluid leaked	23 (4.0)	17 (12.0)	**<0.0001**
	IV became infected	9 (1.6)	1 (0.7)	0.696^x^
	IV fell out	4 (0.7)	5 (3.5)	**0.019**^x^
	Other	21 (3.7)	8 (5.6)	-
**Unsuccessful first attempt PIVC insertion**	**Number of failures**	221 (39.8)^e^	89 (64.0)^f^	**<0.001**^**#**^
**How important do you think it is that we research the best ways to insert, care for and maintain IVs for patients?**	**Median (IQR)**	10 (9,10)^g^	10 (10,10)^h^
*1 = not important; 10 = extremely important*	Not Important (≤ 3)	4 (0.7)	2 (1.4)
	Moderate (4–7)	41 (7.3)	3 (2.1)
	Extremely important (≥ 8)	518 (91.8)	135 (96.4)

Missing data

^a^25

^b^5

^c^28

^d^12

^e^16

^f^3

^g^6

^h^2

Statistical tests

^x^Fisher’s Exact Test

#Chi-square.

Statistically significant results in **bold.**

The respondents’ perception of how well trained the inserter was very similar for both groups (Adult group: median 8, IQR 5, 10; ACC group: median 8, IQR 6, 9; p = 0.44). Likewise, the majority of respondents, both adults and ACCs, believed that it is extremely important to continue to research ways to improve the insertion, care and maintenance of PIVCs.

Further analysis of respondents (adult and adult carers of child) who had greater than 6 PIVCs in the previous 5 years was undertaken to explore this sub-group’s experiences (n = 351). The results regarding insertion were not too dissimilar to the overall study cohort: difficulty with insertion was experienced (median 6.5, IQR 2, 8) and 42.7% identified difficult veins for the reason; and, 90.3% believed that well trained staff are important for successful insertion. In relation to post-insertion care again this group’s responses were consistent with the broader study cohort in that the most common concern with most recent PIVC was pain at the site (22.8%) and the top three complications were pain (15.6%), bruising (13.8%) and swelling (13.1%).

The final question of the survey invited respondents to answer the following open-ended question: Do you have any other comments about your IV therapy experience? This can be in relation to the insertion, care during therapy, or removal. Forty-four percent (313 participants) of respondents chose to include a qualitative comment of their PIVC experience, with 244 responses from adult consumers and 69 responses from ACCs. Responses were then categorised into major themes (Table 4).

**Table 4:** Major themes of respondents regarding their IV therapy experience.

Themes	Subthemes
**Significance of safe and consistent PIV Care**	1. Location of IV
	2. Infection prevention
	3. Inconsistencies
	4. Removal
**Importance of staff training and competence**	1. Technology guided insertion
	2. Skill of inserters is mixed
	3. Safety and comfort
**Value of communication**	1. Previous patient experience
	2. Frustration at not ‘being heard’
	3. Patient education

### Significance of safe and consistent PIV care

This theme incorporated four sub-themes: Location of the PIVC, Infection prevention, Inconsistencies and Removal. Location of the PIVC was referred to as an important concern with the following quotes illustrating this: “It was placed right in the elbow joint region so was painful to bend arm”; “I have become increasing difficult to cannulate … it’s now always in tiny veins, which is painful, causes large bruising and doesn’t last long”; and “Doctors sometimes position it in the wrist … almost every time the wrist is bent it sets off the pump alarm–very annoying”. Decision making of inserters regarding the placement of the PIVC is often driven by the availability of first perceived accessible vein. However, it is evident from these comments that, wherever possible, consideration should also be given to placing the PIVC in a location that does not cause excessive pain nor interfere with the patient’s ability to perform their activities of daily living or their ability to rest and sleep.

The respondents’ comments also revealed that infection prevention is a common concern for patients with a PIVC. Clinical practice standards require health-care professionals to have a clear understanding of the importance of infection prevention practices to prevent complications. It is clear from the following responses that patients also understand this and, at times, were very concerned for their own safety: “I am immunosuppressed so it makes me anxious when staff are lax with hygiene precautions”; “I had staff in a large tertiary facility want to connect my IV line after they had dropped it on the floor”; and “I was so scared every time staff went to give me an antibiotic because they never cleaned the port where the injection went into”.

Patients noticed many inconsistencies in PIVC practices, and their comments reflect greater concern regarding ongoing care rather than with insertion. The following comments illustrate a wide variation of nursing practice at the bedside: “There was great difference between nurses: some had more experience than others in insertion, but biggest difference was with care during therapy”; “Often insertion technique is ok but management / maintenance thereafter is poor”; and “… did not listen to other staff advice nor the guidelines and administered the antibiotic too quickly–this caused great pain and the need for another cannula”. There were however some comments regarding inconsistencies in insertion, as shown by the following example: “Everyone seems to have different ideas about what’s right. There doesn’t seem to be any standard for people putting them in”.

The last sub-theme of comments related to PIVC removal. For consumers removal of the PIVC was as important as the insertion and maintenance phases of the therapy, with the following comments providing evidence for this: “I think the removal is more important, by the time they removed mine my vein was swollen, sore”; “It is annoying that an IV that is working fine has to be taken out after 3 days because finding another vein that doesn’t hurt is difficult”; “IV was left in … and in particular was still in my arm on arriving home (which I did not notice prior to leaving the hospital”; and “Insertion is difficult. Removal can be just as distressing … Last removal = pain ++++ because the nurse tried to pull off transparent dressing while all the tubing was stuck down with other tape. Also she insisted on pressing on the cannula in the vein while she tried to loosen the dressing”.

### Importance of staff training and competence

Three subthemes were contained within this overarching theme: Variable skill of inserters, Technology guided insertion, and Safety and comfort. The first subtheme raised issues around the importance of having standards for inserters so patients feel safe and have trust in health care professionals. The following responses give insight into the importance consumers place on the level of skill of the PIVC inserter and how it influences their experience: “It is hard to assess the training level of staff who insert IVs. You have to place your trust in them that they will maintain standards”; “It is important that the staff are well trained so their IV goes in first try and with limited stress to the child. There is a massive difference in staff trained specifically in paeds [paediatrics]”; and “There needs to be very high levels of skill in emergency and on the wards. Patients need to be examined and if they have difficult veins more experienced personnel need to be called. It is not fun being a guinea pig for inexperienced staff”.

The variable skills of inserters identified by survey participants may have raised the issue of using technology to improve outcomes. The impact of technology on the insertion experience was clearly articulated: “[….] children’s hospital is now using ultrasound in finding a vein. It takes the guess work out of it”; “I have frequent blood tests and infusions with badly damaged veins. On my most recent experience it took medical staff over 20 attempts to insert … My arms were bruised extensively. I think this could have been minimised by using a scanner”; “The best experiences I have had with IV insertion was when the ultrasound machine was used to find a decent vein. Unfortunately these are rarely if ever available to be used on the ward”.

The final subtheme is Safety and comfort, in which many participants linked the importance of training and competence to their own safety and comfort. It is evident from the responses that the participants felt that inadequate inserter competence or training directly impacted on their well-being: “Little concern is given to the patient having multiple attempts and or/failures. It is seen as a patient problem, as though ‘you’ made it fail”; “There were 7 attempts to place the IV …”; and “Most pain and distress I have had was during hospital stays when junior doctors have attempted to cannulate me. I had 21 attempts in 48 hours—it was terrible. More training needed …”

### The Value of communication

The Value of Communication was consistently identified by participants and this theme encompasses: Previous patient experience, Frustration at not being ‘heard’, and Patient education.

The importance of assessing the patient’s previous PIVC experience and preferences was raised: “When I was administered the antibiotics using a syringe directly into the cannula it was always too fast and quite painful—I'd say around a 7–9 on your scale. When I asked about that a nurse was able to use a slower drip feed that did not hurt. However, not all of the nurses used the drip, even when I asked them to. So it would be good for the all of the staff involved in giving care to know what the patient prefers and to do that”; and “My child has a severe fear of needles (has seen a psychologist for it). It is always a traumatic experience. We always advise the staff where the best place to insert IV but they never listen or believe us. Of course they end up in the spot we suggested and in the meantime traumatise our child even further”. The patient’s lived experience of previous PIVCs is an important resource for bedside clinicians.

However, even when patients raised their concerns, their frustration at not being heard is evident: “I could tell (after) about 3 hours that something was wrong, got ignored and then it wasn't working when needed …”; “Staff don't believe you when you tell then it is painful … they prodded and poked at it and said ‘it looks fine’”; “I wish the medical profession would listen to their patients, especially ones like me who have had many hospital admissions over the last 20 years and know their body better than anyone”; and “Listen to me when I say I have experienced difficulty when having cannulas inserted”.

The final subtheme identified is Patient education. The survey participants expressed the importance of health professionals educating them about their PIVC and also the importance of being active participants in their own care: “I think it is VITAL to provide more education to the person getting the cannula”; and “… inform patients on how to correctly care for their IVs e.g. whilst in the shower, correct arm positioning during therapy, and watching out for signs and symptoms of infection/tissuing”.

## Discussion

For many consumers, the experience of PIVC insertion and maintenance is often not a trivial event and can be associated with significant pain, stress and concern. The survey revealed that children experienced more difficult PIVC insertions and insertion-related pain and distress; higher first PIVC insertion attempt failure and, consequently, more insertion attempts; and a higher complication rate than adults. Similarly, older adults (> 65 years) were found to experience greater pain, stress and difficulty with PIV insertion compared with younger adults. Consumers frequently feel their opinions are disregarded when it comes to PIVC insertion and management and, as shown in these survey results, they want to be active participants in their care but are sometimes denied that opportunity.

The findings from the current survey are similar to previously published surveys focusing on patients’ experience of PIVCs. Robinson-Reilly et al. [12] asked 15 patients from two rural oncology units what is was like for them to be repeatedly cannulated. The themes that emerged including feelings of vulnerability; of not having their advice or concerns listened to; and of pain and suffering associated with cannulation. Some patients even reported being more concerned with the PIVC insertion than the chemotherapy. In line with the current survey, patients identified that care could be improved by encouraging collaborative decision-making between the clinician and the patient. Similarly, Larsen et al. [32] found, in a cohort of 10 medical/surgical patients, that these patients reported communication breakdowns between the inserter and themselves and that this was a cause for concern. These patients were keen to suggest areas for improvement, including insertion techniques, insertion site choice and ways to increase comfort and reduce anxiety, which suggests that they are keen to be more active participants in their care.

To address the unacceptably high failure rates, there has been a renewed focus on the methods of insertion and care of PIVCs, and VADs in general, but to date, clinicians and researchers have not concentrated on the consumer as an important partner in reducing PIVC failure and complications. Despite a focus on active consumer engagement by various agencies and in the literature [33], there is too little consumer engagement in policy making and minimal encouragement of active patient participation in care [34]. Similarly, patient-reported outcomes remain absent from a large number of research studies and consumer participation in the research process is limited at best [35]. The aim of active consumer involvement in these areas is to improve service delivery, patient satisfaction and patient outcomes [36]. A Cochrane systematic review [37] assessed the engagement of consumers in developing health care policy and clinical practice guidelines and found that, despite the importance of consumer engagement being widely recognised, little evidence exists regarding levels of consumer participation, how to effectively achieve this, and how to evaluate its success. Rycroft-Malone et al. [38] provide a simple model for practicing evidence-based health care which incorporates research; clinical experience; patients, clients and carers; and local context and environment. The authors claim that by combining these four types of evidence in clinical practice, a focus on patient-centred care is achieved. Recognition of the patient as a key source of evidence in providing person-centred evidence-based care is fundamental to a safe, effective and quality patient-clinician relationship. The authors concede, however, that while patients’ experiences and preferences should be at the core of evidence-based practice, it is unclear to what extent this actually occurs or the contribution it makes to patient outcomes [38].

The survey results indicate the need to include the patient in decisions and care processes to improve outcomes and consumer satisfaction. Consumers want to be more active participants in their care and to be viewed as a valuable resource. However, some of the respondents’ comments reveal that, rather than being seen as important contributors to their care, consumersumers active participants in their care and to be viewed as a valuablInterventions targeted at directly involving patients in the care of their PIVC, the efficacy of which can be directly measured, should be a priority for researchers and clinicians alike. In this way, it can be determined if active consumer participation in PIVC care reduces adverse effects. Effective consumer engagement has been successful in a variety of settings, for example: older adults transitioning across different care facilities [39]; patients with cancer [40]; and in a primary health care setting [41]. When striving to increase such consumer participation in care and shared decision-making, the barriers and facilitators of these aims must be considered. In a systematic review and meta-analysis of the literature, Legare et al. [42] identified the three main barriers to the implementation of shared decision-making as: time constraints, lack of applicability due to patient characteristics, and clinical situation; and the three main facilitators as the motivation of health care professionals in achieving shared decision-making, the perception that shared decision-making will improve patient outcomes, and the perception that shared decision-making will lead to improved health care processes. Further to this, a recent Cochrane review of 39 studies exploring interventions to improve shared decision-making concluded that such interventions appear to be more effective if they target both the patient and the health professional [43].

First insertion success is an important factor in preserving vessel health because the risk of PIVC failure increases for each subsequent catheter insertion [21]. The survey results indicate that 40% of adults and a concerning 64% of children reported experiencing first insertion attempt failure for their most recent PIVC. First attempt PIVC insertion failure rates are reportedly up to 35% for adults [44] and between 50–60% for children [45]; therefore the survey respondents reported higher than documented rates of insertion failure. This is possibly due to recall bias and the non-purposive sampling method used which may lead to an over-representation of respondents who have experienced PIVC problems, but nevertheless the first attempt failure rates are unacceptably high. Both adult consumers and ACCs believed that the use of ultrasound was an important determinant of PIVC insertion success. This is confirmed by recent evidence in which the use of ultrasound was found to improve insertion success, particularly in patients with difficult peripheral venous access [46–49]. In addition to repeated insertion attempts being painful for the patient, this practice is also costly in terms of the extra human and material resources required to achieve successful placement, and therefore the use of ultrasound may also prove to be a cost-saving measure. Both adults and ACCs reported they believed that staff skill in the area of PIVC insertion and adequate hydration were the most important factors in determining insertion success: factors supported by the literature [44, 50, 51]. Use of vein location technology and staff skill in PIVC insertion and care require appropriately trained vascular access clinicians to ensure high quality and safe care.

The levels of pain and stress associated with PIVC insertion revealed by both adult consumers and ACCs should not be disregarded or downplayed by clinicians. Many survey comments suggest that pain and stress are not being seriously considered, with one respondent stating that their PIVC inserter was ‘indifferent’ to her distress. The survey findings confirmed that the pain and stress experienced were significantly higher for children than adults, which is in keeping with the published evidence [9–11]. Additionally, many adult respondents describing their experience of PIVCs identified that insertion was also a source of stress for their families/loved ones. There are many strategies that can be employed by clinicians to minimise the pain and stress that exists around PIVC insertion, including the administration of local anaesthetic. Given the levels of pain and stress identified in the survey, it is important that strategies are used to improve the patient experience of a PIVC. Bond et al.'s [52] systematic review of 37 primary research studies using 17 anaesthetic types concluded that the pain of applying any local anaesthetic is less than that of cannulation without an anaesthesia. Additionally, Griffith et al.’s [53] Cochrane systematic review involving nine studies indicated that the use of vapocoolant spray reduces insertion pain and those studies that included participant satisfaction found the majority of participants would choose pain treatment again. Pain relief for PIVC insertion should be standard practice in adults and children.

Complications are common during PIVC therapy [54], and the survey findings revealed that around a quarter of adults and a third of children with a PIVC experienced complications post-insertion, many of which were potentially preventable and could be mitigated by more active involvement of the patient/family in their care. By encouraging such involvement from patients and families, issues such as inadequate dressing and securement, handwashing prior to handling PIVCs, proper disinfection of the hub and pain or redness at the PIVC site could be identified early, which may ultimately lead to a decrease in PIVC failure and complications. Indeed, Weingart et al. [25] found that active consumer participation in care led to a decrease in adverse events. They found that patients who were active in their care were able to ‘observe, identify and communicate potential problems before they resulted in medical injuries’. Respondents in this survey reported concerning clinical practices that clearly contravene infection prevention protocols. Consumers with a PIVC should feel able to speak up when witnessing poor practice. By encouraging the active involvement of consumers in their own PIVC care, substandard practice could be identified and prevented from occurring. Similarly, given the priority to remove redundant PIVCs to minimise complication risks, the consumer could be a useful resource if empowered to question why the PIVC remains in situ.

There were many comments from respondents highlighting poor practice around infection prevention during PIVC insertion and maintenance. This is in alignment with a recent case study from the United Kingdom authored by a hospitalised consumer, who is also an infection prevention professional [55], which highlighted poor infection prevention practices, including infrequent disinfection of the PIVC hub prior to use and poor PIVC dressings which left the insertion site exposed. Consumers are very aware of many components of infection prevention, particularly hand washing and disinfection of the insertion site, and they identified many breaches in these areas. They perceived this to be a threat to their safety, a lack of care on the behalf of the clinician, and an area that needs to be greatly improved. It is evident from the survey that consumers felt that more can be done to improve the care of PIVCs, with the overwhelming majority of both adults and ACCs stating that ongoing research into the insertion, care and maintenance of PIVCs was extremely important.

Demonstrating some respondents’ feelings of lack of involvement and active participation in their care are comments regarding the clinician’s failure to listen to their previous experiences regarding PIVC success and failure. Many respondents directed the inserter to the most reliable veins and/or cannulation sites prior to PIVC insertion based on their numerous previous experiences, but they reportedly were not listened to, and in many instances insertion failure subsequently occurred. Current international guidelines already include a focus on determining consumer preferences in decisions about their own care, including IV device site and selection [56–60]. In Australia, the National Safety and Quality Health Service Standards prioritise partnering with consumers in care decisions [27]. However, it would appear from the survey results that much more needs to be done to integrate consumer preferences into routine practice. Given the importance of achieving PIVC insertion on the first attempt in preserving vessel health, listening to the patient and including them in site selection wherever possible would be a useful strategy. Bedside decision tools for PIVC insertion should include prompts to seek patient preferences in device and site selection before any cannulation attempts, where possible. Routine PIVC assessment should incorporate prompts for staff to ask patients about any concerns with the PIVC and respond accordingly.

Given that PIVC consumers appear to want to be more active participants in their care, any intervention aimed at achieving this must also target clinicians and policy makers who drive workplace culture. A successful example of such an intervention is the educational model employed to increase hand hygiene compliance amongst health care workers in the United Kingdom [61–63]. This model used interventions targeted at both consumers and health care workers in an effort to address poor hand hygiene practices and, through empowering patients to take responsibility for their own health and ensuring that their voices were heard and acted upon, the model was successful in increasing hand washing by an average of 50% [63]. Similarly, a nationwide multimodal hand hygiene culture-change initiative was launched by the Australian Commission on Safety and Quality in Health Care which actively engaged consumers in the cultural change and saw hand hygiene rates increase from 43.6% to 67.8% over the first two years of implementation [64].

The main strength of this study is that it has allowed consumers the opportunity to share their experiences and recommendations for PIVC practice change with policy makers, clinicians and researchers. The limitations of this study include the potential for bias that is inherent in self-reporting. Specifically, those with negative experiences may have been more likely to respond to the survey and contribute remarks and comments, and that recall bias (where the survey respondents may not accurately recall events or experiences from the past) may have influenced the survey results. The inability to calculate response rates, given the nature of the survey distribution, means that the degree of sampling bias is unknown and thus the results cannot be generalised to all patients receiving IV catheterisation. Additionally, the cross-sectional, non-purposive sampling technique used in the survey where the majority of the participants were from Australia indicates that the results cannot be generalised across the entire cohort of consumers with a PIVC as our sample may not be a true representation of the cohort.

## Conclusions

This survey confirms that PIVC insertion, maintenance and removal can be a painful, concerning, and stressful experience for consumers, and one which they feel needs to be improved and align with best practice guidelines for PIVC insertion and care. The results highlight the importance of providing appropriately educated and trained vascular access clinicians to ensure best practice and high quality care. Children and older consumers report a worse PIVC experience than younger adults. Specific strategies to improve the consumer experience of PIVC insertion and care include: pain relief for PIVC insertion as standard practice; decision-making tools which include patient preference in PIVC site selection; and PIVC daily assessment tools which encourage patient input. Consumer engagement is important to improving outcomes and providing person-centred evidence-based care. Future practice, research and translation of research should focus on outcomes and strategies important to consumers and on ways to improve their participation and engagement in care: listening to them and taking action to minimise pain, complications and repeated attempts would be a good first step.
